# Context dependency and saturating effects of loss of rare soil microbes on plant productivity

**DOI:** 10.3389/fpls.2015.00485

**Published:** 2015-06-30

**Authors:** W. H. Gera Hol, Wietse de Boer, Mattias de Hollander, Eiko E. Kuramae, Annelein Meisner, Wim H. van der Putten

**Affiliations:** ^1^Department of Terrestrial Ecology, Netherlands Institute of Ecology, WageningenNetherlands; ^2^Department of Microbial Ecology, Netherlands Institute of Ecology, WageningenNetherlands; ^3^Department of Soil Quality, Wageningen University, WageningenNetherlands; ^4^Microbial Ecology Group, Department of Biology, Lund University, LundSweden; ^5^Center for Macroecology, Evolution, and Climate, Natural History Museum of Denmark, University of Copenhagen, CopenhagenDenmark; ^6^Department of Biology, University of Copenhagen, CopenhagenDenmark; ^7^Laboratory of Nematology, Wageningen University, WageningenNetherlands

**Keywords:** plant productivity, land use, rare biosphere, species loss, dilution-to-extinction

## Abstract

Land use intensification is associated with loss of biodiversity and altered ecosystem functioning. Until now most studies on the relationship between biodiversity and ecosystem functioning focused on random loss of species, while loss of rare species that usually are the first to disappear received less attention. Here we test if the effect of rare microbial species loss on plant productivity depends on the origin of the microbial soil community. Soils were sampled from three land use types at two farms. Microbial communities with increasing loss of rare species were created by inoculating sterilized soils with serially diluted soil suspensions. After 8 months of incubation, the effects of the different soil communities on abiotic soil properties, soil processes, microbial community composition, and plant productivity was measured. Dilution treatments resulted in increasing species loss, which was in relation to abundance of bacteria in the original field soil, without affecting most of the other soil parameters and processes. Microbial species loss affected plant biomass positively, negatively or not at all, depending on soil origin, but not on land use history. Even within fields the effects of dilution on plant biomass varied between replicates, suggesting heterogeneity in microbial community composition. The effects of medium and severe species loss on plant biomass were similar, pointing toward a saturating effect of species loss. We conclude that changes in the composition of the soil microbial community, including rare species loss, can affect plant productivity, depending on the composition of the initial microbial community. Future work on the relation between function and species loss effects should address this variation by including multiple sampling origins.

## Introduction

The demands for increasing production of biomass for food, feed, and bioenergy production have led globally to progressing land use expansion and intensification (Foley et al., 2005). Land use intensification has been associated with biodiversity loss (Foley et al., 2011; Tsiafouli et al., 2015), increased greenhouse gas emissions (De Vries et al., 2013), and loss of biological pest control (Tscharntke et al., 2012). The causal relations between those three factors are unclear, but in general biodiversity is associated with enhanced ecosystem functioning (Balvanera et al., 2006). The positive relation between diversity and function is predominantly based on random species loss scenarios, and has been primarily addressed with plant communities. The use of randomly assembled communities of mostly abundant species may have limited power to accurately predict consequences of species loss for ecosystem functioning, since in reality species will not get lost at random (Zavaleta and Hulvey, 2004).

The risk of species to get lost depends on their sensitivity to the disturbance but also on their abundance. It has been shown that rare species are at higher risk of going extinct than abundant species (Gaston, 2008). The consequences of loss of rare species might be negligible, if species contributions to function are in relation to their abundance (The Mass Ratio hypothesis, Grime, 1998). However, rare species contribute most to phylogenetic diversity (Mi et al., 2012) and may have unique functions (Mouillot et al., 2013). Hence it is important to determine their effects on ecosystem functioning. Besides that, it is also important to determine if rare species influence ecosystem functioning independent of the land use intensity of the ecosystem considered.

Land use differences may generate variation in microbial community composition (Nacke et al., 2011). Intensive land use leads to overall lower diversity with an increased chance of pathogen accumulation (Kremen and Miles, 2012). In low intensity managed soils microbial diversity is expected to be higher and mutualistic microbes could play a more prominent role in nutrient acquisition. Therefore, consequences of microbial species loss might be opposite in low intensity managed soil compared to intensive arable soils. In addition, soils from different land use types will vary in properties such as organic matter which could influence consequences of species loss as well (Philippot et al., 2013).

Studying consequences of microbial species loss from soil started over 30 years ago with the serial dilution approach first applied by Salonius (1981). The aim of this approach is to create soils with a range of microbial diversities. This can be achieved by inoculation of sterilized soil with dilutions from a soil suspension. The dilution will result in loss of species, especially those of low abundance. An incubation period will allow the microbial biomass to recover to equal amounts in all inoculated soils. Such experimental treatments enable testing the effect of varying microbial community composition on functions like nitrogen cycling (Philippot et al., 2013), decomposition (Baumann et al., 2013), antifungal volatile production (Hol et al., 2015), and resistance to invasions from pathogens (van Elsas et al., 2012; Mallon et al., 2015), but it has rarely been done for plant productivity. Species removal studies used mostly soil from one location, like pasture (Griffiths et al., 2001; Wertz et al., 2006, 2007; Philippot et al., 2013) or arable land (Griffiths et al., 2004; Hol et al., 2010). Few studies, if any, have considered effects of species loss in soils with different land use, such as low versus high intensity used soils, on plant productivity. Therefore, we tested consequences of microbial diversity loss for primary productivity in relation to land use type.

The aim of the present study was to test if the effect of rare microbial species loss on plant productivity depends on the origin of the microbial soil community. We used sterilized soil inoculated with serial dilutions (Salonius, 1981) to examine the consequence of rare microbial species loss from 12 different soils from three land use types: intensive arable, rotation arable, and permanent pasture. We test the hypothesis that the effect of loss of microbes on plant productivity depends on soil origin. The underlying assumption is that the relative abundance of beneficial and pathogenic microbes will vary between soils from different land use types.

## Experimental Procedures

### Soil Sampling and Sterilization

In July 2009, soil was collected from two farms in Sweden. On each farm three fields were sampled that differ in land use: intensive wheat rotation (arable intensive), crop rotation (arable rotation), and permanent grassland (pasture). The pasture soil was not tilled during the last 10 years. The arable rotation included winter wheat, with grass present in a 5 years rotation and kept for at least a year. The arable intensive soil was used for annual crops, including winter wheat and spring barley, with annual tillage. Soil characteristics of fields are described by De Vries et al. (2013). Duplicate samples (36 kg) were taken from each field, separated by minimally 30 m. In the end there were 12 soil origins (2 farms × 3 land use types × 2 replicates per field). The soil was sieved over 4 mm diameter to remove stones and roots and distributed over six plastic bags each containing 6 kg of sieved soil. For each sampling location, five bags were sterilized by γ-radiation (>25 kGray) at ISOTRON, Ede, The Netherlands, and one bag was stored at 4°C to be used as living inoculum. Two times 50 g soil from each soil origin was used for inoculum (see next paragraph). The bag with inoculum for one soil origin (Farm two Arable rotation field replicate two) was accidentally sterilized and hence the sterilized soil from this origin was inoculated with a soil suspension from the other field replicate (Farm two Arable rotation field replicate one). Subsamples from the inoculum soils were stored at -20°C for molecular analyses.

### Dilution, Inoculation, and Incubation

In order to create soils with increasing severity of microbial species loss, γ -sterilized soil was inoculated with a gradient of diluted soil suspensions according to Hol et al. (2010) with minor modifications. The 12 soil origins were used for the creation of 12 soil suspensions. Each suspension was obtained by placing 62.5 g fresh soil in a Waring blender with 200 ml sterilized demineralized water. The material was blended five times for 1 min at high speed. In-between the blender was cooled on ice for 2 min, to prevent overheating of the blender and microbes. The aim of the blending was to release as many microbes as possible from the soil particles into the solution. The suspension was then transferred to four Greiner tubes of 50 ml and centrifuged for 10 min at 1000 × *g* at 4°C. Afterward the tubes were decanted to remove the soil particles and the supernatant was poured over a 45 μm sieve to remove soil fauna such as nematodes. The suspension is then used to create in total three dilution treatments. The highest inoculum concentration was 1:200, i.e., 5 kg fresh weight (fw) sterile soil received a microbial suspension (200 ml) based on 25 g fw field soil. We mixed 2 ml of the 1:200 suspension with 198 ml sterilized demineralized water to create the 1:20000 dilution and repeated the inoculation procedure described above. This 1:20000 suspension was used to inoculate two bags of 5 kg each and two ml was used for the last dilution step, 1:2000000. Bags were closed with a cotton plug and a rubber band, to allow some air diffusion but prevent contamination by airborne microbes. After inoculation, the bags were closed and manually shaken to mix the suspension with the sterilized soil. The bags were incubated in the dark at room temperature for 8 months, to allow colonization of the sterilized soil and regrowth of the microbial communities to comparable microbial biomass in the different dilutions. Bags with the inoculated soils were mixed biweekly by shaking and turning the bags. Bags were weighted to check moisture content, which was adjusted to 15% water content (w/w) when necessary.

### Assessments After the Incubation Period

After the incubation period it was crucial to determine whether microbial biomass and abiotic parameters were comparable between dilution treatments; else these parameters could be important confounding factors. Soil was sampled after the 8 month incubation period from the 36 bags (2 farms × 3 field × 2 replicates per field × 3 dilutions). Microbial biomass was determined by collecting four samples of 10 g dw soil from each bag: two for fumigation and two for direct extraction (Vance et al., 1987). For biomass estimates of bacteria and fungi, the phospholipid fatty acids (PLFAs) in soil were analyzed according to Boschker (2004) with a Bligh and Dyer extraction. Approximately 2 g freeze-dried dry soil was used per sample, with two technical replicates for each of the 36 (2 farms × 3 fields × 2 replicates per field × 3 dilutions) treatments. As markers for bacterial PLFA’s we took the sum of i15:0, a15:0, 15:0, i16:0, 16:1w9, i17:0, a17:0, cy17:0, 18:1w7, cy19:0 (Frostegård and Bååth, 1996), and as fungal marker 18:2w6,9 (Federle, 1986).

For analysis of protozoan biomass, hundred g fresh soil was used to check whether protozoa numbers recovered from dilution. Flagellates and amoebae numbers were estimated by a modified most probable number (MPN) method (Rønn et al., 1995). Briefly, three grams of homogenized soil were shaken vigorously in 30 ml amoebae saline solution for 20 min. Three-fold dilution series of the soil slurries were performed in 96 well microtiter plates with 0.3 g l^-1^ tryptic soy broth (TSB, Difco) as growth media. Plates were incubated at 15°C and inspected by inverted microscopy for the presence of protozoa after one and 3 weeks.

To assess whether species loss would affect the potential N mineralization rate, the transformation of arginine into ammonium was measured (Arginine Ammonification; Alef and Kleiner, 1986; Lin and Brookes, 1999). Of each bag, five subsamples of fresh soil (based on 1 g dry soil) were weighted in a Greiner 15 ml tube. Three subsamples received 0.25 ml arginine solution (1 × 10^-2^g L-arginine ml^-1^ MilliQ water) and two unamended subsamples served as background controls. The arginine concentration added was determined based on a pilot with two extreme soils. All tubes were mixed by vortexing and incubated for 3 h at 25°C. After incubation 4 × 10^-3^ L 2M KCl was added and tubes were shaken for 0.25 h to extract NH_4_^+^ from soil. After shaking the background control subsamples also received 0.25 ml arginine solution. All tubes were centrifuged at 5250 × *g* for 10 min after which 0.7 ml supernatant was transferred to an Eppendorf tube which already contained 0.7 ml MilliQ water to dilute to 1 M KCl for analyses. Samples were stored at -20°C until N-NH_4_^+^ concentrations were measured on a Technicon TrAAcs 800 autoanalyser (Technicon Instruments Corp., Tarrytown, NY., USA). The differences in ammonium concentrations between treated and background control tubes were taken as ammonification potential.

### Experimental Setup

We tested if the effects of microbial species loss on plant growth interacted in soils with a history of different land use by installing 36 treatments (2 farms × 3 land use types × 2 replicates per field × 3 dilutions). The two bags of soil for each dilution treatment were mixed at the end of the incubation period before filling pots (see previous paragraph). For each treatment, 24 pots were filled with moist soil based on 200 g dw soil resulting in 864 pots in total. Each pot was sown with one seed of winter wheat *Triticum aestivum* cv. Carenius. Pots were placed in a fully randomized block design with 24 blocks in a greenhouse under 60% relative humidity; 16 h L, 8 h D, 21°C/16°C, and additional illumination by 400 W growing bulbs (Philips SONT-T Agro, Philips, Eindhoven, The Netherlands). Light intensity at plant level was 225 μmol PAR. Each block contained one replicate of all 36 treatments. Pots without seedlings were re-sown. In the end 37 pots had no seedlings. Pots without seedlings were found mostly for soils from farm 1 (35 of the 37 empty pots), but within this farm the absent seedlings occurred across all dilution treatments and land use types. Plants were watered regularly, approximately 25 ml of demineralized water three times a week without providing additional nutrients. After 8 weeks growth during spring, plants were harvested by clipping the shoots at soil surface, and drying them at 70°C until constant weight. From a subset of 72 pots (6 blocks × 1 farm × 2 land use types × 3 dilutions) roots were stored in 50% EtOH to check for arbuscular mycorrhizal fungi (AMF) colonization by staining (Vierheilig et al., 1998). Interestingly, no AMF colonization was observed in any of the roots. Apparently the preparation of the soil suspension was unfavorable to AMF, as AMF do occur in the field soil (De Vries et al., 2013; Tsiafouli et al., 2015). From the 72 pots 2 g soil was stored at -20°C for DNA extraction and amplification by PCR.

### Molecular Analyses of the Bacterial Community

The bacterial community was analyzed with multiplex pyrosequencing of 16S rRNA partial gene to determine whether dilution led to species loss and shifts in bacterial community composition. Dilution will most likely also have affected archaeal, fungal, and protozoan community composition. We therefore refer to effects of dilution as effects of microbial species loss and use the bacterial analysis to validate that dilution causes species loss. The pyrosequencing analysis was done for two soil origins with different land use history (arable intensive and pasture) that showed opposite reactions in plant growth to loss of rare microbes. DNA was extracted from 0.25 g soil with Mobio PowerSoil DNA Extraction kit (Mo Bio Laboratories Inc., Carlsbad, CA, USA) according to the manufacturer’s protocol. Forty four samples in total were analyzed: two samples from the original field soil, six samples after incubation, and 36 samples collected at the end of the experiment. DNA was amplified in a 2 × 50 μl PCR reaction with the primer set 515F 806R (Lauber et al., 2009) targeting the V4 region of the bacterial 16S rRNA gene. The 515F primer included the Roche 454-B pyro-sequencing adapter and GT linker, while 806R included the Roche 454-A sequencing adapter and GG linker. A 12-bp barcode was included in both primers. After PCR 2 μl product was checked by agarose gel electrophoresis. The two reactions per sample were pooled and purified with the QIAquick PCR purification kit (Qiagen, Valencia, CA, USA). Twenty MIDs were used in total, with 10 different MIDs per pyro-sequencing lane. The samples were sequenced on a Roche 454 automated sequencer and GS FLX system using titanium chemistry (454 Life Sciences, Branford, CT, USA; Macrogen Inc. Company, South Korea). Sequence analysis was done according to Navarrete et al. (2013). Briefly, only sequences which had a limited mismatch to the primer sequences and barcodes, contained no homopolymer run exceeding six nucleotides and showed no ambiguous characters were assigned to samples. Denoising and chimera removal were done with Denoiser 0.91 (Reeder and Knight, 2010) and UCHIME version 4.2.40 (Edgar et al., 2011). Sequences within the range of 300–380 bp were accepted and bases with a quality score lower than 25 were trimmed. Data was clustered into Operational Taxonomic Units (OTUs) with the program UCLUST version 1.2.21 (Edgar, 2010) using a cutoff of 97% similarity. The most dominant OTU within a cluster was identified using the Ribosomal Database Project (RDP) 2 classifier (release 10.4), with a minimum support threshold of 60%. In total 425268 high quality sequences were obtained, on average 5595 per sample, with a minimum of 1456 and a maximum of 16228. Samples which are compared were always rarefied to the same number of sequences using the “vegan” package in R 3.0.3 (R Core Development Team, 2014). The relative abundance of OTUs (reads per OTU/total reads) was used to calculate Shannon’s Index H = -1Σ*p*ln*p* (Shannon and Weaver, 1949) and eveness E_H_ = H/lnS (Pielou, 1975).

### Statistical analyses

Technical replicates were averaged before further analysis. The samples collected directly after incubation were tested for overall dilution effects across all soil origins. The non-parametric Friedman rank sum test was used to test for differences between dilution treatments across all 12 soil origins for soil characteristics (pH, NO_3_^-^, NH_4_^+^, P) and microbial biomass data (microbial carbon, protozoan number, PLFA) since transformation of the data did not remove the violations of normality. The 12 soil origins were treated as blocking factor and the three dilution treatments formed the groups. Significant tests were followed by Wilcoxon paired rank tests between dilution treatments, still using the 12 soil origins as blocks.

Estimates of species loss after dilution were made by comparing OTUs in the field soil with OTU in the soil at the end of the experiment. The correlation between species loss and relative abundance in field soil was tested with Pearson correlation tests, for the relative abundances that were represented by five or more OTUs. Differences in species loss between dilution treatments were tested with Wilcoxon paired rank tests, using relative abundance for pairing.

Shoot biomass data was analyzed with a linear mixed effects model, using dilutions, and land use as fixed factors. We used a split-plot design where: block (24 blocks in the greenhouse) was considered as main plot; farm as its subplot; land use as subplot of farm; field replicate as subplot of land use; and dilution as subplot of field replicate. Re-sown and empty pots were not included in the analyses of shoot biomass. Normality of errors was checked by plotting residuals against fitted values and testing against the normal distribution with Kolmogorov–Smirnov tests. Despite moderate violations of normality, the result of the linear mixed effects model was consistent with non-parametric tests (Kruskal–Wallis followed by Wilcoxon) and therefore we show the results from the linear mixed effects model. The significance of the interaction between dilution and land use was determined by comparing the two models (with and without interaction) with each other using ANOVA. Since the interactive effects between dilution and land use were significant, and also within land use the soil origins showed a significant interaction with dilution treatment, the analysis of significant dilution treatment effects was done separately for each soil origin: linear mixed effects model with dilution as fixed factor and block as random factor, using the sharpened Benjamini, and Hochberg procedure to correct the *P*-value for the multiple comparisons (Benjamini and Hochberg, 2000; Verhoeven et al., 2005).

In order to determine whether the effect of dilution on shoot biomass within a soil origin was consistent, we calculated the differences in average shoot biomass between dilution treatments and plotted those against each other. The slope of this correlation would indicate whether the effect of the 10^6^ treatment was similar to the effect of 10^4^ treatment (slope = 1), whether there was an additional effect of further species loss (slope>1) or whether the effect of the 10^6^ treatment was less than the 10^4^ treatment (slope<1). The strength of the correlation was calculated with Spearman Rank Correlation test. The intercept and slope of the correlation was estimated with Siegel repeated medians method, which calculates the slope between all points and selects the median as estimate. To test the significance of the association between the treatment differences, a Monte Carlo permutation test was used: shoot biomass values were randomly assigned to a dilution treatment within soil origin after which Spearman rank correlation coefficients were calculated and slopes were estimated. The correlation coefficient and slope of the actual shoot biomass data was compared with the total distribution of correlation coefficients based on permuted datasets (*n* = 10000). The analyses were performed with R 3.0.3 (R Core Development Team, 2014) using packages ‘nlme’, ‘pspearman,’ and ‘stats.’

## Results

### Dilution Treatment Effects on Soil Characteristics

After 8 months of incubation, the estimators of microbial abundance (microbial carbon, bacterial, and fungal PLFA, number of protozoa) and most abiotic parameters (except NO_3_^-^), and N mineralization were not different among dilution treatments. Differences in microbial and abiotic parameters between soils from different land use types were consistent, with pasture soils a magnitude different from the arable soils (**Table 1**).

**Table 1:** Microbial biomass (microbial carbon, number of protozoa, PLFA), chemical soil characteristics (concentrations of NO_3_^-^, NH_4_^+^, P_olson_, and pH) and N mineralization potential (arginine ammonification) with dilution treatments after 8 months incubation (average ± SEM per land use, *n* = 4).

		Dilution			P*
		10^2^	10^4^	10^6^	
Microbial carbon	Intensive arable	257 ± 32	276 ± 27	282 ± 16	0.11
(mg C kg^-1^ soil)	Rotation arable	320 ± 20	351 ± 5	343 ± 23	
	Pasture	409 ± 39	459 ± 43	441 ± 42	
# protozoa	Intensive arable	0.6 ± 0.3	1.5 ± 1.0	1.6 ± 1.2	0.06
(10^5^ g^-1^ soil)	Rotation arable	1.8 ± 0.1	4.6 ± 1.4	4.4 ± 1.2	
	Pasture	5.5 ± 2.0	15.3 ± 8.4	16.1 ± 7.7	
Bacterial PLFA	Intensive arable	1.5 ± 0.4	1.8 ± 0.4	1.6 ± 0.4	0.78
(μg g^-1^ soil)	Rotation arable	1.2 ± 0.5	1.2 ± 0.4	1.5 ± 0.3	
	Pasture	4.2 ± 1.4	4.8 ± 1.5	5.8 ± 1.8	
Fungal PLFA	Intensive arable	3.1 ± 0.5	2.9 ± 0.2	4.1 ± 1.7	0.92
(10^-2^ μg g^-1^ soil)	Rotation arable	3.3 ± 1.1	2.9 ± 1.3	3.2 ± 1.1	
	Pasture	7.8 ± 1.6	9.2 ± 2.8	11.2 ± 3.5	
NO_3_^-^ (mg kg^-1^)	Intensive arable	120 ± 8	111 ± 13	117 ± 8	0.04
	Rotation arable	101 ± 16	89 ± 9	92 ± 13	
	Pasture	363 ± 12	320 ± 30	315 ± 22	
NH_4_^+^ (mg kg^-1^)	Intensive arable	3.7 ± 0.4	3.8 ± 0.5	5.1 ± 0.7	0.05
	Rotation arable	4.3 ± 0.2	4.4 ± 0.2	4.7 ± 1.0	
	Pasture	8.3 ± 0.9	9.5 ± 1.0	8.9 ± 1.6	
P_olson_	Intensive arable	50 ± 5	50 ± 6	50 ± 6	0.78
(mg kg^-1^)	Rotation arable	41 ± 8	40 ± 8	42 ± 8	
	Pasture	125 ± 35	129 ± 37	126 ± 36	
pH H_2_O	Intensive arable	6.5 ± 0.7	6.6 ± 0.7	6.5 ± 0.7	0.11
	Rotation arable	5.6 ± 0.5	5.6 ± 0.5	5.6 ± 0.5	
	Pasture	6.7 ± 0.5	6.8 ± 0.5	6.8 ± 0.5	
pH KCl	Intensive arable	6.2 ± 0.8	6.3 ± 0.8	6.2 ± 0.8	0.45
	Rotation arable	5.1 ± 0.5	5.1 ± 0.5	5.1 ± 0.5	
	Pasture	6.7 ± 0.6	6.8 ± 0.5	6.9 ± 0.5	
Arginine	Intensive arable	133 ± 54	137 ± 57	237 ± 143	0.92
Ammonification	Rotation arable	18 ± 30	3 ± 48	55 ± 25	
(μg g^-1^ hr^-1^)	Pasture	225 ± 43	241 ± 56	218 ± 34	

P*, P-value based on Friedman rank sum test, testing for differences between dilution treatments across all 12 soil origins (2 farms × 3 land use types × 2 replicates per field).

### Dilution Treatment Effects on Soil Bacteria

**FIGURE 1:**
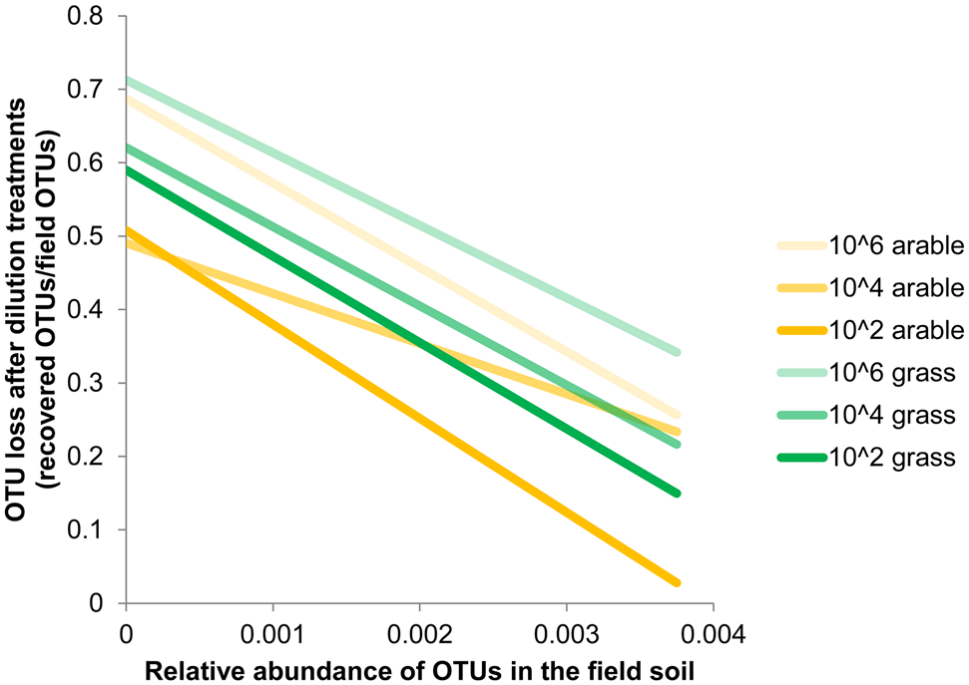
**Loss of Operational Taxonomic Units (OTUs) after dilution treatments correlates with abundance of OTUs in the field.** Estimated by comparing OTUs detected at the end of the greenhouse experiment with the relative abundance in the pasture and arable field soil. Bacterial OTUs were obtained after 454-pyrosequencing of 16S rRNA gene fragments from field soil (*n* = 1) and the soil from the dilution treatments after plant growth (*n* = 5 (pasture) or *n* = 6 (arable) samples with 2000 reads per sample). Relative abundance is the number of reads per OTU/total number of OTUs. Species loss for each relative abundance was calculated as: (the number of field OTUs that were not detected in the greenhouse experiments / total number of field OTUs). Trend lines based on least squares are shown, underlying data is presented in Supplementary Table S2.

Species richness decreased severely after the dilution treatment and incubation period, in comparison to the field soil (Supplementary Table S1A), but partly recovered during the greenhouse experiment in the presence of plant roots (Supplementary Table S1B). The bacteria in the field soils show a typical distribution with few dominant and many low abundant species (Supplementary Table S2). The five most dominant bacterial species in both field soils were found in all dilution treatments at the end of the plant growth phase (Supplementary Table S2). However, many OTUs with low abundance in the field soil were not detected at the end of the experiment. The number of non-detected OTUs in experimental soil versus total OTUs in field soil (i.e., ‘OTU loss’) correlated negatively with relative abundance (**Figure 1**, Pearson correlation: Pasture *df* = 16, *P* < 0.002, Arable *df* = 10, *P* < 0.04). In both soils, increasing dilution lead to significantly increasing species loss (Wilcoxon paired rank test comparing dilution treatments 10^4^ and 10^6^ with 10^2^ Pasture *n* = 18, *P* < 0.045, Arable *n* = 12, *P* < 0.008). The number of OTUs was reduced with each dilution step, while at higher taxonomic levels (phyla) only in the most diluted treatment a decline was observed (**Table 2**). The effects of dilution treatment varied slightly between the two soils, with Shannon H and evenness E*_H_* decreasing earlier with dilution in the pasture soil but eventually reaching lowest values in the arable soil in the most diluted treatment (**Table 2**).

**Table 2:** Dilution and incubation effects on soil bacterial community composition in pasture and intensive arable soil.

		Phyla richness	OTU richness S	Shannon H	Evenness E_H_
Pasture	10^2^	13.7 ± 0.3a	647 ± 19a	5.92 ± 0.05a	0.91 ± 0.003a
	10^4^	13.6 ± 0.6a	583 ± 22b	5.59 ± 0.11b	0.88 ± 0.012b
	10^6^	11.8 ± 0.6b	496 ± 15c	5.45 ± 0.05b	0.88 ± 0.003b
Arable	10^2^	13.3 ± 0.6a	607 ± 25a	5.81 ± 0.05a	0.91 ± 0.003a
	10^4^	12.5 ± 0.4a	578 ± 11b	5.71 ± 0.02a	0.90 ± 0.001a
	10^6^	10.8 ± 0.3b	443 ± 11c	5.25 ± 0.05b	0.86 ± 0.005b

*Data based on 454-pyrosequencing of bacterial amplicons of 16S rRNA gene fragments from bulk soil. Sampling depth is 2000 per sample, *n* = 5–6 per treatment, Operational Taxonomic Units (OTUs) cut-off is 97%. Different letters indicate significant differences between dilutions within a soil origin.*

The phylogenetic distribution of the bacteria was affected strongly by the incubation period without plants; severe drops in relative abundance were observed for the Acidobacteria, Nitrospira, WS3, and also the unknown bacteria (Supplementary Table S3). The period of plant growth increased the share of the Acidobacteria, TM7, and the Verrucomicrobia (Supplementary Table S3).

### Dilution Treatment Effects on Plant Biomass

**FIGURE 2:**
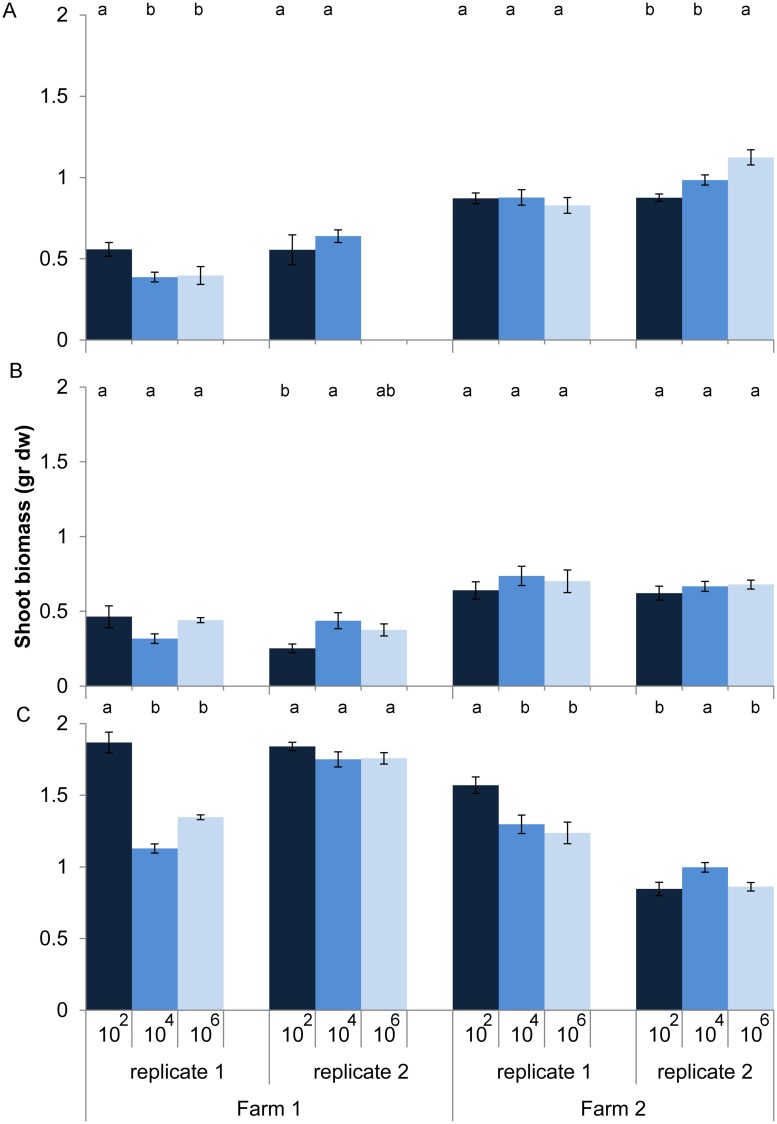
**Shoot biomass (average ± SEM) of *Triticum aestivum* after 8 weeks of growth in sterilized soils inoculated with diluted soil suspensions from two farms and three land use types: **(A)** arable intensive, **(B)** arable rotation, and **(C)** pasture**. The color of the bars represent the dilution treatment, with dark blue = 10^2^ (least diluted inoculums), blue = 10^4^ and light blue = 10^6^ (most diluted inoculums). Significant differences between dilution treatments within soil origin are indicated by different letters.

Effects of loss of microbes on plant growth depended on land use (dilution*land use, *P* < 0.001, anova, L. ratio = 44.14, *df* m1 = 15, m2 = 11). Within land use there was still a significant interaction between dilution and soil origin. Zooming in on the individual soil origins shows that for half of the 12 soil origins microbial species loss increased or decreased shoot biomass without any clear relationship with land use, while for the other six soil origins no effect of microbial species loss on shoot biomass was observed (**Figure 2**). In soil from pastures the expectation that microbial species loss would lead to a decline in plant growth was confirmed in only two out of four cases, while plants growing in arable soils were overall not responding to species loss (**Figure 2**).

**FIGURE 3:**
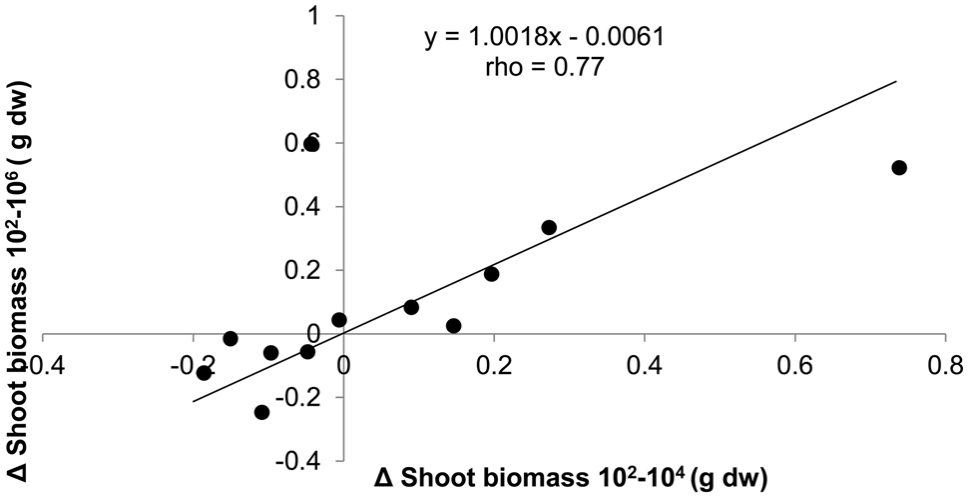
**The absolute differences in shoot biomass of *T. aestivum* between dilution treatments plotted against each other for each of the 12 soil origins.** Spearman rank correlation test *n* = 12, *P* = 0.004.

Since the effect of dilution on plant biomass was positive, negative, or neutral depending on soil origin, we tested the consistency of dilution treatment effects within soil origin to explore whether it could have been a purely stochastic process. This appeared not to be the case: when shoot biomass in the 10^4^ treatment was smaller than biomass in the 10^2^, then the same applied to shoot biomass in the 10^6^, as shown by the significant positive correlation between treatment differences (**Figure 3**). Comparison with permuted data show that the chance to obtain such a high correlation and slope is relatively small (*P* = 0.039). The positive correlation with slope close to 1 and an intercept close to 0 shows that most effect on shoot biomass is observed with the first species loss event, which is from 10^2^ to 10^4^, and virtually no additional effects with further species loss from the 10^6^ treatment. The same approach was taken to look for consistency of dilution effects within a field, by calculating the correlation between dilution effects in field replicate one with field replicate two for all six fields. No significant association between the dilution effects of the field replicates was found (slope = 0.20, rho = 0.47, *P* = 0.13).

## Discussion

Our results do not provide support for the hypothesis that the effect of microbial species loss on plant growth would depend on land use type. Instead, we observed high variation in plant response to the dilution treatments. Plant biomass responded positively, negatively or not at all to microbial species loss. As results of dilution treatments on plant biomass were not consistent across all samples, other hitherto unknown factors may have determined re-colonization and functioning of the soil microbial communities.

The high variation in plant response to dilution probably reflects variation in the microbial community used as inoculum. Even plants growing in soil inoculated with soil microbial communities originating from the same field showed divergent responses; only in rotational arable soil from farm two the replicates showed the same response to dilution, but these were both inoculated with suspensions from exactly the same soil origin. One source of variation could be unpredictable community assembly during recolonization of the soil (Pagaling et al., 2014), but the similar response of suspensions 10^4^ and 10^6^ from the same origin suggests that this is not the case. Those results could be seen as idiosyncratic, in the sense that each soil origin has its own reaction to dilution (Hol et al., 2011). The variation in response to dilution from samples originating from the same field could be caused by spatial variation in microbial community composition. Franklin and Mills (2003) showed spatial heterogeneity in microbial community composition in an arable field, with spatial autocorrelations up to 6 m. Hol et al. (2010) collected inoculum from an intensively managed arable field with 1 m between samples and this resulted in consistent responses to dilution. In the current experiment at least 30 m separated the different inoculum collection sites. The >30 m distances between samples may have caused the large variation in plant response to dilution, probably due to differences in the abundances of pathogens and mutualists within the microbial communities.

Another factor in the variation in effects of microbial species loss could be an interaction with abiotic conditions. The soils varied widely among land use types in nitrate and available phosphorus, and this may have affected the scope for plant-microbe interactions. For instance, Davitt et al. (2011) showed plant-microbe symbioses to be more beneficial under resource limiting conditions. However, we observed that replicates within a field showed minor variation in their abiotic characteristics (data underlying **Table 1**), yet the plants growing on those soils generally showed different reactions to dilution (**Figure 2**). Hence, the differences in microbial community composition appear to cause more variation than interactions with abiotic conditions.

The dilution method was successful in reducing species richness, and most severe species loss was observed for the low abundant species. Given the concerns about the quantitative accuracy of pyrosequencing, it is rather surprising to see the strong correlation between relative abundance and species loss. The sequencing depth of this study is relatively superficial, and will underestimate total species loss. Future studies should apply deeper sampling to get a better view of the identity and potential function of the species with abundances that were currently below detection limits. While the data show that the dilution approach is effective in removing species and especially those of lower abundances, it also shows that shifts occurred in the microbial community composition. In theory the rank order should be similar between treatments, in practice it may vary as a result of disturbed species interactions and differences in growth rate during recolonization (Tardy et al., 2014). In our study the dominant phylum stayed the same in all dilution treatments (Proteobacteria), but in other phyla and at lower taxonomic levels shifts were observed. Therefore, the effect of microbial species loss on plant biomass production could be the direct effect of absence of species, or an indirect effect from the shift in abundance of species. The experimental setup with the incubation period will be vulnerable to priority effects (Fukami et al., 2010), but also in real-life species loss events the indirect effects such as shifts in abundances of the remaining species have to be taken into account.

For the arable intensive soil, increase of plant growth after dilution was expected, based on presence of pathogens (Kremen and Miles, 2012) and the results from an earlier species loss study in intensively managed arable soil (Hol et al., 2010). Positive effects of dilution were found once in the arable intensive soil, but neutral, and negative effects occurred as well. It is possible that dilution effects were underestimated by only measuring shoot biomass; an earlier study found the belowground part to be most responsive (Hol et al., 2010). Yet, the positive or negative effects of species loss on plant productivity demonstrates that microbial community composition plays a modifying role on plant biomass production, which is opposite to the idea of a consistent positive relationship between soil microbial species richness and plant productivity (Seneviratne and Kulasooriya, 2013). Plants on arable rotation soils were rather unresponsive to species loss, which may have to do with abiotic constraints, but further study is required before it can be concluded that plants in rotation soils have less dependencies on microbes. Diluted soils from fields with permanent pasture reduced plant biomass in half of the cases, indicating that at least in some cases loss of beneficial microbes occurred. The benefits could be direct, by promotion of plant growth via nutrition or indirect via suppression of pathogens.

Although care is needed to extrapolate results from highly controlled experiments to larger scales, the occurrence of a severe species loss event with a regrowth period shares similarities with intensively managed systems that apply soil treatments such as fumigation or steam sterilization. Those systems will be recolonized by the few surviving species or from the surroundings, which could easily lead to rapid invasions by pathogens. For such systems further research into suitable inocula to obtain stable microbial community and higher plant productivity would be beneficial. Our choice of study species most likely will give a conservative estimate for the role of microbial diversity for plant growth in general. Forbs and legumes are known to be more strongly affected by microbial diversity than grasses (Wagg et al., 2014). It is premature to discuss potential consequences of rare microbial species loss in more natural surroundings with mixed plant communities since all work on microbial species loss thusfar only used single plants per pot. Plant competition can influence the sensitivity of plant to microbial feedback (Hol et al., 2013) and soil biota may change the competition between plant species (Van der Putten et al., 1993). Currently it is unknown to what extent plant-soil feedback interactions may depend on low abundant microbial species.

Our finding that the effects of microbial species loss could vary depending on the initial microbial community could have implications for other dilution studies. Most studies used only one or few soil origins and thus may provide a biased or relatively incomplete picture of the responses of functioning to species loss. When the function of interest depends on species with contrasting effects and those species have different spatial distributions, then soil origin could be highly influential. We found that variation between soil origins even within close distance were as important as variations in land use history. However, less variations between soil origins is expected for those processes that are based on species richness, such as resource competition, and niche preemption preventing invasion (Mallon et al., 2015). Ultimately, with the advancement of technology and knowledge of plant-microbe interactions, measurements of soil biodiversity should allow a risk analysis of disturbance factors impacting biodiversity and the consequences for plant productivity.

## Author Contributions

WH, WB, and WP design the study. WH, AM collected the data. WH, AM, MH, EK analyzed the data. WH, AM, EK, WP interpreted the data. WH wrote the first draft and all co-authors contributed substantially to revisions.

## Conflict of Interest Statement

The authors declare that the research was conducted in the absence of any commercial or financial relationships that could be construed as a potential conflict of interest.
