# Supplementary material for: Context dependency and saturating effects of loss of rare soil microbes on plant productivity
Source: Front Plant Sci. 2015 Jun 30;6:485. doi: 10.3389/fpls.2015.00485 (PMC4485053; doi:10.3389/fpls.2015.00485)
Supplement: Supplementary file 1 [file Table_S1-S3.docx]

**Table S1a** Dilution and incubation effects on soil bacterial community composition in pasture and intensive arable soil. Data based on 454-pyrosequencing of bacterial amplicons of 16S rRNA gene fragments from bulk soil. Sampling depth is 1456 reads per sample, n=1 per treatment, replication for rarefaction = 3, OTUs cut-off is 97%.

|  |  |  | Phyla  Richness | OTU  richness  S | Shannon  H | Evenness  E_H_ |
| --- | --- | --- | --- | --- | --- | --- |
| Pasture | Field soil |  | 13.3±0.7 | 642±9 | 6.08±0.02 | 0.94±0.001 |
|  | Incubation | 10^2^ | 11.3±0.3 | 457±3 | 5.50±0.01 | 0.90±0.001 |
|  | Incubation | 10^4^ | 12±0.6 | 487±5 | 5.55±0.01 | 0.90±0.001 |
|  | Incubation | 10^6^ | 11.7±0.3 | 400±6 | 5.19±0.02 | 0.87±0.001 |
|  |  |  |  |  |  |  |
| Arable | Field soil |  | 14.7±0.6 | 663±2 | 6.14±0.01 | 0.94±0.001 |
|  | Incubation | 10^2^ | 11.7±0.7 | 434±4 | 5.21±0.02 | 0.86±0.001 |
|  | Incubation | 10^4^ | 10.7±0.3 | 315±3 | 4.62±0.01 | 0.80±0.001 |
|  | Incubation | 10^6^ | 11±0 | 287±0 | 4.31±0 | 0.76±0 |

**Table S1b** Dilution and incubation effects on soil bacterial community composition in pasture and intensive arable soil at the end of the greenhouse experiment. Similar to table 2 in the main text, but rarefied to 1456 to allow comparison to Table S1a. Novel OTUs are OTUs not detected in field soil. Unique OTUs are OTUs only detected in a particular dilution treatment. Data based on 454-pyrosequencing of bacterial amplicons of 16S rRNA gene fragments from bulk soil. Sampling depth is 1456 reads per sample, n=5-6 per treatment, replication for rarefaction = 1, OTUs cut-off is 97%.

|  |  |  | Phyla  Richness | OTU  richness  S | Shannon  H | Evenness  E_H_ | Novel  OTUs | Unique OTUs |
| --- | --- | --- | --- | --- | --- | --- | --- | --- |
|  | Greenhouse | 10^2^ | 14.2±0.7 | 562±14 | 5.84±0.04 | 0.90±0.003 | 387±12 | 22±2 |
| Pasture | Greenhouse | 10^4^ | 12.8±0.6 | 488±16 | 5.49±0.1 | 0.89±0.011 | 334±12 | 14±3 |
|  | Greenhouse | 10^6^ | 11.4±0.4 | 426±10 | 5.39±0.04 | 0.89±0.004 | 305±5 | 10±1 |
|  |  |  |  |  |  |  |  |  |
|  | Greenhouse | 10^2^ | 12±0.3 | 526±18 | 5.72±0.05 | 0.91±0.004 | 337±14 | 9±2 |
| Arable | Greenhouse | 10^4^ | 11.5±0.4 | 485±8 | 5.62±0.02 | 0.91±0.001 | 317±5 | 4±1 |
|  | Greenhouse | 10^6^ | 9.8±0.3 | 385±5 | 5.21±0.03 | 0.87±0.004 | 252±5 | 3±1 |

Table S2a Low abundance bacterial species are more vulnerable to species loss following dilution and incubation of a grassland soil.

| Field soil |  |  | Greenhouse soil: recovered OTUs^3^ | | | | Greenhouse soil: % species loss^4^ | | |
| --- | --- | --- | --- | --- | --- | --- | --- | --- | --- |
| Relative abundance^1^ | Number of OTUs ^2^ | | 10^2 | 10^4 | 10^6 |  | 10^2 | 10^4 | 10^6 |
| 0.00017 | 430 |  | 185 | 180 | 134 |  | 0.57 | 0.58 | 0.69 |
| 0.00035 | 234 |  | 109 | 114 | 85 |  | 0.53 | 0.51 | 0.64 |
| 0.00052 | 135 |  | 65 | 51 | 45 |  | 0.52 | 0.62 | 0.67 |
| 0.00070 | 91 |  | 43 | 40 | 30 |  | 0.53 | 0.56 | 0.67 |
| 0.00087 | 57 |  | 30 | 26 | 20 |  | 0.47 | 0.54 | 0.65 |
| 0.00105 | 50 |  | 34 | 29 | 23 |  | 0.32 | 0.42 | 0.54 |
| 0.00122 | 26 |  | 13 | 11 | 8 |  | 0.50 | 0.58 | 0.69 |
| 0.00139 | 29 |  | 15 | 17 | 16 |  | 0.48 | 0.41 | 0.45 |
| 0.00157 | 19 |  | 11 | 11 | 6 |  | 0.42 | 0.42 | 0.68 |
| 0.00174 | 21 |  | 13 | 13 | 11 |  | 0.38 | 0.38 | 0.48 |
| 0.00192 | 16 |  | 12 | 9 | 9 |  | 0.25 | 0.44 | 0.44 |
| 0.00209 | 13 |  | 8 | 7 | 7 |  | 0.38 | 0.46 | 0.46 |
| 0.00227 | 8 |  | 4 | 4 | 3 |  | 0.50 | 0.50 | 0.63 |
| 0.00244 | 11 |  | 6 | 7 | 6 |  | 0.45 | 0.36 | 0.45 |
| 0.00261 | 6 |  | 4 | 4 | 2 |  | 0.33 | 0.33 | 0.67 |
| 0.00279 | 7 |  | 6 | 4 | 4 |  | 0.14 | 0.43 | 0.43 |
| 0.00296 | 5 |  | 4 | 3 | 3 |  | 0.20 | 0.40 | 0.40 |
| 0.00314 | 8 |  | 7 | 8 | 6 |  | 0.13 | 0.00 | 0.25 |
| 0.00331 | 2 |  | 1 | 2 | 1 |  | 0.50 | 0.00 | 0.50 |
| 0.00349 | 1 |  | 1 | 1 | 1 |  | 0.00 | 0.00 | 0.00 |
| 0.00383 | 2 |  | 2 | 2 | 1 |  | 0.00 | 0.00 | 0.50 |
| 0.00401 | 1 |  | 1 | 1 | 1 |  | 0.00 | 0.00 | 0.00 |
| 0.00418 | 3 |  | 1 | 1 | 0 |  | 0.67 | 0.67 | 1.00 |
| 0.00436 | 1 |  | 1 | 1 | 1 |  | 0.00 | 0.00 | 0.00 |
| 0.00453 | 1 |  | 1 | 1 | 1 |  | 0.00 | 0.00 | 0.00 |
| 0.00471 | 1 |  | 1 | 1 | 0 |  | 0.00 | 0.00 | 1.00 |
| 0.00488 | 2 |  | 1 | 1 | 1 |  | 0.50 | 0.50 | 0.50 |
| 0.00505 | 1 |  | 1 | 1 | 1 |  | 0.00 | 0.00 | 0.00 |
| 0.00523 | 1 |  | 1 | 1 | 0 |  | 0.00 | 0.00 | 1.00 |
| 0.00540 | 4 |  | 4 | 4 | 3 |  | 0.00 | 0.00 | 0.25 |
| 0.00593 | 1 |  | 1 | 1 | 1 |  | 0.00 | 0.00 | 0.00 |
| 0.00610 | 3 |  | 3 | 3 | 2 |  | 0.00 | 0.00 | 0.33 |
| 0.00627 | 3 |  | 2 | 3 | 3 |  | 0.33 | 0.00 | 0.00 |
| 0.00645 | 1 |  | 1 | 1 | 0 |  | 0.00 | 0.00 | 1.00 |
| 0.00662 | 1 |  | 1 | 1 | 1 |  | 0.00 | 0.00 | 0.00 |
| 0.00680 | 1 |  | 1 | 1 | 1 |  | 0.00 | 0.00 | 0.00 |
| 0.00697 | 2 |  | 1 | 1 | 1 |  | 0.50 | 0.50 | 0.50 |
| 0.00715 | 1 |  | 1 | 1 | 0 |  | 0.00 | 0.00 | 1.00 |
| 0.00732 | 1 |  | 1 | 1 | 1 |  | 0.00 | 0.00 | 0.00 |
| 0.00749 | 1 |  | 1 | 1 | 1 |  | 0.00 | 0.00 | 0.00 |
| 0.00819 | 1 |  | 1 | 1 | 1 |  | 0.00 | 0.00 | 0.00 |
| 0.00837 | 2 |  | 1 | 1 | 1 |  | 0.50 | 0.50 | 0.50 |
| 0.00941 | 1 |  | 0 | 1 | 0 |  | 1.00 | 0.00 | 1.00 |
| 0.00959 | 1 |  | 1 | 1 | 1 |  | 0.00 | 0.00 | 0.00 |
| 0.01290 | 1 |  | 1 | 1 | 1 |  | 0.00 | 0.00 | 0.00 |
| 0.01359 | 1 |  | 1 | 1 | 1 |  | 0.00 | 0.00 | 0.00 |
| 0.01499 | 1 |  | 1 | 1 | 1 |  | 0.00 | 0.00 | 0.00 |
| 0.01725 | 1 |  | 1 | 1 | 1 |  | 0.00 | 0.00 | 0.00 |

1. Relative abundance = number of reads per OTU/ divided by the total number of reads for the field soil. The lowest abundance 0.00017 equals 1 read, n=5738 for the field soil from the grassland.
2. The number of OTUs in the field soil is shown for each abundance class. E.g. the grassland field soil contained 430 singletons.
3. These 3 columns indicate for each dilution treatment the number of OTUs from a particular abundance class in the field that was detected at the end of the greenhouse experiment. E.g. from the 430 singletons in the field soil only 134 were detected in the most diluted treatment while this was 185 for the least diluted treatment. The estimates for the recovered OTUs are all based on rarefied data, n=2000.
4. Here species loss is shown, for each abundance class calculated as 1-(greenhouse detected OTUs/field OTUs). E.g. for 10^6^ for the lowest abundance the species loss is calculated as 1-(134/430) = 0.69. The colors in the columns indicate the severity of species loss. Cells with black borders indicate species loss 0.8-1; this is only found when the number of OTUs per abundance class is low (3 or less).

Table S2b Low abundance bacterial species are more vulnerable to species loss following dilution and incubation of an arable soil.

| Field soil |  |  | Greenhouse soil: recovered OTUs | | | | Greenhouse soil: % species loss | | |
| --- | --- | --- | --- | --- | --- | --- | --- | --- | --- |
| Relative abundance^1^ | Number of OTUs^2^ | | 10^2 | 10^4 | 10^6 |  | 10^2 | 10^4 | 10^6 |
| 0.00031 | 450 |  | 242 | 216 | 157 |  | 0.46 | 0.52 | 0.65 |
| 0.00063 | 210 |  | 120 | 106 | 81 |  | 0.43 | 0.5 | 0.61 |
| 0.00094 | 110 |  | 67 | 61 | 49 |  | 0.39 | 0.45 | 0.55 |
| 0.00125 | 51 |  | 34 | 29 | 22 |  | 0.33 | 0.43 | 0.57 |
| 0.00156 | 37 |  | 26 | 25 | 17 |  | 0.3 | 0.32 | 0.54 |
| 0.00188 | 34 |  | 26 | 26 | 21 |  | 0.24 | 0.24 | 0.38 |
| 0.00219 | 18 |  | 12 | 10 | 8 |  | 0.33 | 0.44 | 0.56 |
| 0.00250 | 18 |  | 15 | 14 | 9 |  | 0.17 | 0.22 | 0.5 |
| 0.00281 | 11 |  | 9 | 8 | 7 |  | 0.18 | 0.27 | 0.36 |
| 0.00313 | 9 |  | 9 | 8 | 9 |  | 0 | 0.11 | 0 |
| 0.00344 | 7 |  | 6 | 5 | 5 |  | 0.14 | 0.29 | 0.29 |
| 0.00375 | 7 |  | 7 | 4 | 4 |  | 0 | 0.43 | 0.43 |
| 0.00406 | 4 |  | 3 | 4 | 3 |  | 0.25 | 0 | 0.25 |
| 0.00438 | 4 |  | 3 | 4 | 4 |  | 0.25 | 0 | 0 |
| 0.00469 | 3 |  | 3 | 3 | 2 |  | 0 | 0 | 0.33 |
| 0.00500 | 4 |  | 2 | 3 | 3 |  | 0.5 | 0.25 | 0.25 |
| 0.00531 | 4 |  | 4 | 4 | 3 |  | 0 | 0 | 0.25 |
| 0.00563 | 3 |  | 3 | 2 | 2 |  | 0 | 0.33 | 0.33 |
| 0.00594 | 1 |  | 1 | 1 | 1 |  | 0 | 0 | 0 |
| 0.00625 | 1 |  | 1 | 1 | 1 |  | 0 | 0 | 0 |
| 0.00656 | 2 |  | 2 | 1 | 1 |  | 0 | 0.5 | 0.5 |
| 0.00688 | 3 |  | 2 | 2 | 1 |  | 0.33 | 0.33 | 0.67 |
| 0.00750 | 1 |  | 1 | 1 | 0 |  | 0 | 0 | 1 |
| 0.00813 | 1 |  | 1 | 1 | 0 |  | 0 | 0 | 1 |
| 0.00875 | 3 |  | 3 | 3 | 3 |  | 0 | 0 | 0 |
| 0.00907 | 1 |  | 1 | 1 | 1 |  | 0 | 0 | 0 |
| 0.00969 | 1 |  | 1 | 1 | 1 |  | 0 | 0 | 0 |
| 0.01188 | 1 |  | 1 | 1 | 1 |  | 0 | 0 | 0 |
| 0.02126 | 1 |  | 1 | 1 | 1 |  | 0 | 0 | 0 |

1. Relative abundance = number of reads per OTU/ divided by the total number of reads for the field soil. The lowest abundance 0.00031 equals 1 read, n=3199 for the field soil from the arable field.
2. The number of OTUs in the field soil is shown, see Table S2a
3. These 3 columns indicate for each dilution treatment the number of OTUs from a particular abundance class in the field that was detected at the end of the greenhouse experiment, see Table S2a for an example
4. Species loss is shown, for each abundance class calculated as 1-(greenhouse detected OTUs/field OTUs). see Table S2a for an example

Table S3a Distribution of phyla for a grassland soil: in the field, after dilution and incubation, and after a greenhouse experiment with *T. aestivum*.

| \|  \| field soil \| incubation \| incubation \| incubation \| greenhouse experiment \| greenhouse experiment \| greenhouse experiment \| \| --- \| --- \| --- \| --- \| --- \| --- \| --- \| --- \| \| Phyla \| undiluted \| 10^2 \| 10^4 \| 10^6 \| 10^2 \| 10^4 \| 10^6 \| \| Proteobacteria \| 39.35% \| 58.17% \| 44.02% \| 53.30% \| 45.15% \| 46.00% \| 42.57% \| \| unknown \| 20.67% \| 8.93% \| 17.93% \| 10.51% \| 12.52% \| 14.56% \| 13.93% \| \| Acidobacteria \| 11.26% \| 3.30% \| 3.57% \| 3.09% \| 5.79% \| 4.18% \| 4.96% \| \| Actinobacteria \| 11.26% \| 8.31% \| 5.49% \| 10.65% \| 13.34% \| 13.57% \| 8.04% \| \| Bacteroidetes \| 7.14% \| 11.26% \| 19.09% \| 9.00% \| 10.43% \| 11.03% \| 12.62% \| \| Verrucomicrobia \| 3.02% \| 1.65% \| 2.82% \| 3.64% \| 3.42% \| 3.71% \| 8.01% \| \| Planctomycetes \| 2.47% \| 2.54% \| 2.40% \| 6.18% \| 4.73% \| 3.10% \| 6.09% \| \| Firmicutes \| 2.13% \| 1.44% \| 0.76% \| 0.48% \| 1.02% \| 0.85% \| 0.33% \| \| Nitrospira \| 1.37% \| 0.00% \| 0.07% \| 0.00% \| 0.06% \| 0.04% \| 0.00% \| \| Gemmatimonadetes \| 0.55% \| 3.78% \| 3.23% \| 2.54% \| 1.88% \| 1.83% \| 2.09% \| \| Crenarchaeota \| 0.27% \| 0.00% \| 0.00% \| 0.07% \| 0.35% \| 0.07% \| 0.01% \| \| WS3 \| 0.27% \| 0.07% \| 0.14% \| 0.00% \| 0.00% \| 0.00% \| 0.00% \| \| Chloroflexi \| 0.07% \| 0.41% \| 0.27% \| 0.41% \| 0.65% \| 0.62% \| 0.37% \| \| OD1 \| 0.07% \| 0.00% \| 0.14% \| 0.07% \| 0.10% \| 0.03% \| 0.84% \| \| BRC1 \| 0.00% \| 0.00% \| 0.07% \| 0.00% \| 0.07% \| 0.11% \| 0.05% \| \| Chlamydiae \| 0.00% \| 0.00% \| 0.00% \| 0.07% \| 0.03% \| 0.19% \| 0.00% \| \| Cyanobacteria \| 0.00% \| 0.00% \| 0.00% \| 0.00% \| 0.10% \| 0.00% \| 0.00% \| \| OP10 \| 0.00% \| 0.00% \| 0.00% \| 0.00% \| 0.01% \| 0.01% \| 0.00% \| \| Spirochaetes \| 0.00% \| 0.14% \| 0.00% \| 0.00% \| 0.00% \| 0.00% \| 0.00% \| \| TM7 \| 0.00% \| 0.00% \| 0.00% \| 0.00% \| 0.34% \| 0.10% \| 0.10% \| |  |  |  |  |  |  |  |  |  |  |  |  |  |  |
| --- | --- | --- | --- | --- | --- | --- | --- | --- | --- | --- | --- | --- | --- | --- | --- | --- | --- | --- | --- | --- | --- | --- | --- | --- | --- | --- | --- | --- | --- | --- | --- | --- | --- | --- | --- | --- | --- | --- | --- | --- | --- | --- | --- | --- | --- | --- | --- | --- | --- | --- | --- | --- | --- | --- | --- | --- | --- | --- | --- | --- | --- | --- | --- | --- | --- | --- | --- | --- | --- | --- | --- | --- | --- | --- | --- | --- | --- | --- | --- | --- | --- | --- | --- | --- | --- | --- | --- | --- | --- | --- | --- | --- | --- | --- | --- | --- | --- | --- | --- | --- | --- | --- | --- | --- | --- | --- | --- | --- | --- | --- | --- | --- | --- | --- | --- | --- | --- | --- | --- | --- | --- | --- | --- | --- | --- | --- | --- | --- | --- | --- | --- | --- | --- | --- | --- | --- | --- | --- | --- | --- | --- | --- | --- | --- | --- | --- | --- | --- | --- | --- | --- | --- | --- | --- | --- | --- | --- | --- | --- | --- | --- | --- | --- | --- | --- | --- | --- | --- | --- | --- | --- | --- | --- | --- | --- | --- | --- | --- | --- | --- | --- | --- | --- | --- | --- | --- | --- | --- | --- | --- |
|  |  |  |  |  |  |  |  |  |  |  |  |  |  |  |
|  |  |  |  |  |  |  |  |  |  |  |  |  |  |  |

| Table S3b Distribution of phyla for an arable soil: in the field, after dilution and incubation, and after a greenhouse experiment with *T. aestivum*. |  |  |  |  |  |  |  |  |  |  |  |  |  |  |
| --- | --- | --- | --- | --- | --- | --- | --- | --- | --- | --- | --- | --- | --- | --- |
|  |  |  |  |  |  |  |  |  |  |  |  |  |  |  |
|  |  |  |  |  |  |  |  |  |  |  |  |  |  |  |
| \|  \| field soil \| incubation \| incubation \| incubation \| greenhouse experiment \| greenhouse experiment \| greenhouse experiment \| \| --- \| --- \| --- \| --- \| --- \| --- \| --- \| --- \| \| Phyla \| undiluted \| 10^2 \| 10^4 \| 10^6 \| 10^2 \| 10^4 \| 10^6 \| \| Proteobacteria \| 37.77% \| 44.16% \| 31.66% \| 17.38% \| 35.43% \| 40.20% \| 43.48% \| \| unknown \| 18.20% \| 8.45% \| 6.52% \| 8.04% \| 12.61% \| 12.37% \| 11.45% \| \| Acidobacteria \| 12.84% \| 3.78% \| 1.44% \| 1.30% \| 5.39% \| 4.12% \| 2.40% \| \| Actinobacteria \| 13.05% \| 28.71% \| 45.33% \| 59.82% \| 23.94% \| 19.62% \| 21.97% \| \| Bacteroidetes \| 7.90% \| 7.76% \| 10.71% \| 10.10% \| 10.14% \| 10.97% \| 10.28% \| \| Verrucomicrobia \| 3.23% \| 2.20% \| 1.65% \| 0.89% \| 5.17% \| 5.93% \| 5.78% \| \| Planctomycetes \| 2.61% \| 1.72% \| 0.27% \| 0.27% \| 3.29% \| 3.15% \| 2.11% \| \| Firmicutes \| 0.89% \| 0.55% \| 0.21% \| 0.21% \| 0.32% \| 0.33% \| 0.23% \| \| Nitrospira \| 0.89% \| 0.21% \| 0.07% \| 0.21% \| 0.01% \| 0.07% \| 0.00% \| \| Gemmatimonadetes \| 1.30% \| 2.13% \| 1.92% \| 0.76% \| 1.92% \| 2.43% \| 1.37% \| \| Crenarchaeota \| 0.55% \| 0.00% \| 0.00% \| 0.21% \| 0.16% \| 0.19% \| 0.08% \| \| WS3 \| 0.14% \| 0.00% \| 0.00% \| 0.00% \| 0.00% \| 0.00% \| 0.00% \| \| Chloroflexi \| 0.34% \| 0.34% \| 0.21% \| 0.82% \| 1.01% \| 0.45% \| 0.85% \| \| OD1 \| 0.07% \| 0.00% \| 0.00% \| 0.00% \| 0.01% \| 0.09% \| 0.00% \| \| BRC1 \| 0.00% \| 0.00% \| 0.00% \| 0.00% \| 0.02% \| 0.02% \| 0.00% \| \| Chlamydiae \| 0.00% \| 0.00% \| 0.00% \| 0.00% \| 0.00% \| 0.00% \| 0.00% \| \| Cyanobacteria \| 0.14% \| 0.00% \| 0.00% \| 0.00% \| 0.40% \| 0.00% \| 0.01% \| \| OP10 \| 0.07% \| 0.00% \| 0.00% \| 0.00% \| 0.11% \| 0.01% \| 0.00% \| \| Spirochaetes \| 0.00% \| 0.00% \| 0.00% \| 0.00% \| 0.00% \| 0.00% \| 0.00% \| \| TM7 \| 0.00% \| 0.00% \| 0.00% \| 0.00% \| 0.06% \| 0.05% \| 0.00% \| |  |  |  |  |  |  |  |  |  |  |  |  |  |  |
|  |  |  |  |  |  |  |  |  |  |  |  |  |  |  |
|  |  |  |  |  |  |  |  |  |  |  |  |  |  |  |
